# Sensitivity to lunar cycles prior to the 2007 eruption of Ruapehu volcano

**DOI:** 10.1038/s41598-018-19307-z

**Published:** 2018-01-24

**Authors:** Társilo Girona, Christian Huber, Corentin Caudron

**Affiliations:** 1 Department of Earth, Environmental and Planetary Sciences, Brown University, Providence, Rhode Island USA; 2 Département Géosciences, Environnement et Société, Université Libre de Bruxelles (ULB), Brussels, Belgium; 3 Department of Seismology and Gravimetry, Royal Observatory of Belgium, Uccle, Belgium; 4 Present Address: Jet Propulsion Laboratory, California Institute of Technology, Pasadena, CA 91109 USA; 5 Present Address: Department of Geology, Ghent University, Krijgslaan 281 (S8-WE13), Ghent, Belgium

## Abstract

A long-standing question in Earth Science is the extent to which seismic and volcanic activity can be regulated by tidal stresses, a repeatable and predictable external excitation induced by the Moon-Sun gravitational force. Fortnightly tides, a ~14-day amplitude modulation of the daily tidal stresses that is associated to lunar cycles, have been suggested to affect volcano dynamics. However, previous studies found contradictory results and remain mostly inconclusive. Here we study how fortnightly tides have affected Ruapehu volcano (New Zealand) from 2004 to 2016 by analysing the rolling correlation between lunar cycles and seismic amplitude recorded close to the crater. The long-term (~1-year) correlation is found to increase significantly (up to confidence level of 5-sigma) during the ~3 months preceding the 2007 phreatic eruption of Ruapehu, thus revealing that the volcano is sensitive to fortnightly tides when it is prone to explode. We show through a mechanistic model that the real-time monitoring of seismic sensitivity to lunar cycles may help to detect the clogging of active volcanic vents, and thus to better forecast phreatic volcanic eruptions.

## Introduction

The possibility that Moon-Sun gravitational forces can influence terrestrial volcanism has been widely debated over the last century^1–21^. The most intriguing debate lies on whether fortnightly tides, a ~14-day amplitude modulation of the daily tidal stresses that is related to lunar phases, affect volcanic activity or even force eruptions to occur some specific days instead of others. For example, *Johnston and Mauk*^22^ suggested that major eruptions at Stromboli (Italy) start preferentially close to the fortnightly tidal minima (neap tides), that is, when the moon phase is close to the first or third quarter. The same correlation with neap tides was reported for five of the six dome extrusions occurring between 1879 and 1880 in Islas Quemadas volcano^23^ (El Salvador). In contrast, *Sottili and Palladino*^24^ suggested that the frequency of small explosive events in Stromboli increases during fortnightly tidal maxima (spring tides), that is, close to full or new moon. The same correlation with spring tides was reported for Kilauea (Hawai’i, US), Fuego (Guatemala), Ngauruhoe (New Zealand), and Mayon (Philippines) volcanoes^25–28^. On the other hand, fortnightly tidal modulation does not apparently affect the onset of eruptions at Mauna Loa^25,29^ (Hawai’i).

**Figure 1:**
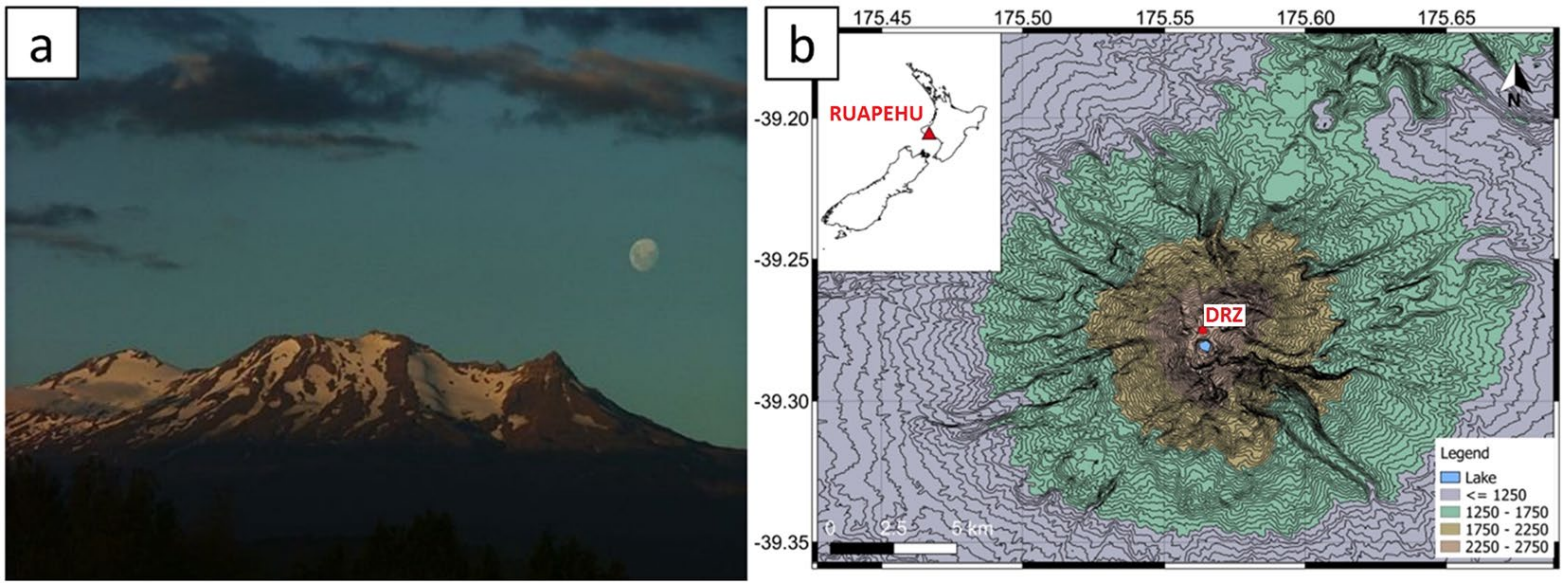
Ruapehu volcano. (**a**) Photo of Mt Ruapehu with the Moon in the background; by courtesy of Greg Steenbeeke. (**b)** Location of Mt Ruapehu and the GeoNet seismic station (DRZ) used in this study. This map was generated with QGIS 2.16.1 (http://www.qgis.org/en/site/), contour lines were extracted from the ASTER Global Digital Elevation Model (GDEM) (ASTER GDEM is a product of NASA and METI; https://asterweb.jpl.nasa.gov/gdem.asp), latitude and longitude are given in degrees, and altitude in legend is given in meters.

The aforementioned studies appear to yield contradictory conclusions as to the nature or existence of correlation between lunar phases (i.e., fortnightly tidal modulation) and volcanic activity. This may be a statistical bias due to the unknown start time of many historical eruptions, and therefore to the small number of events considered^30^. To overcome these limitations, we analyse whether fortnightly tides affect volcanoes by addressing the following questions: is the persistent seismicity recorded around active volcanic centres sensitive to fortnightly tidal modulation? If so, can we use this sensitivity to detect when a volcano is in a critical state and prone to erupt? We tackle these questions by exploring the correlation between lunar phases and seismic amplitude (hereafter called luni-seismic correlation) at Ruapehu volcano, New Zealand (Fig. 1). In particular, we use data from a seismic station installed at the summit of Ruapehu because: (a) the processes that are more likely to respond to tidal forcing are expected to take place at shallow levels beneath the volcanic crater^5,31^, and (b) nearly-continuous data are openly available for the last 13 years (we use data from 22-February-2004 to 15-November-2016, Geonet-GNS archive, DRZ station, vertical component^32^). Ruapehu is a good candidate for this study as it has displayed a broad spectrum of behaviour over the last decade, including several episodes of unrest, periods of quiescence, a small gas explosion (October 4, 2006), and a large phreatic eruption which occurred without warning^32,33^ (September 25, 2007).

The analysis we undertake here consists of five main steps. First, we compute the logarithm of the daily seismic amplitude *ln*(*y*_*sam*_) from the raw seismic data, after applying a 20-day high-pass median filter to remove the influence of potential processes occurring over timescales greater than the average periodicity of spring tides, *T* = 14.7653 days (i.e., the average time between full moon and the next new moon) (Supplementary Fig. S1). Second, we create a synthetic periodic time series to parameterize lunar cycles: *y*_*lun*_ = −*cos*(2*π*(*t* − *t*_*low*_)/*T*), where *t* is time in days and *t*_*low*_ is a reference day of the calendar with neap tide (i.e., quarter moon). With this approach, *y*_*lun*_ = 1 during high tides (full or new moon) and *y*_*lun*_ = −1 during low tides (quarter moon) (Supplementary Fig. S2). Third, we calculate the Pearson product-moment correlation coefficient (*ρ*) among different subsets of the time series *ln*(*y*_*sam*_) and *y*_*lun*_ (Supplementary Fig. S3). We use backward windows of 1 year (i.e., >300 data pairs capturing more than 20 spring/neap tides), which allow us to explore the existence of long-term luni-seismic correlation and preserves a statically relevant sample size; the analysis is also performed with backward windows from 100 to 600 days for the sake of comparison. The Pearson coefficient *ρ* evaluates if two datasets are linearly correlated, such that 0 < *ρ* ≤ 1 implies a positive correlation between *ln*(*y*_*sam*_) and *y*_*lun*_, while −1 ≤ *ρ* < 0 implies a negative correlation; the magnitude of *ρ* indicates the strength of the correlation between the time series. Fourth, we test if *ρ* obtained for every moving window is significantly different from zero by calculating the probability *p*_*value*_ of obtaining by chance the observed (or more extreme) values of *ρ*. Fifth, we combine a set of Monte Carlo simulations with a binomial test to analyse the likelihood for a stochastic seismic amplitude time series to produce the correlation coefficients observed in the natural data (Supplementary Fig. S4). A detailed explanation of the data processing and analysis can be found in Methods (Sections 1 and 2).

**Figure 2:**
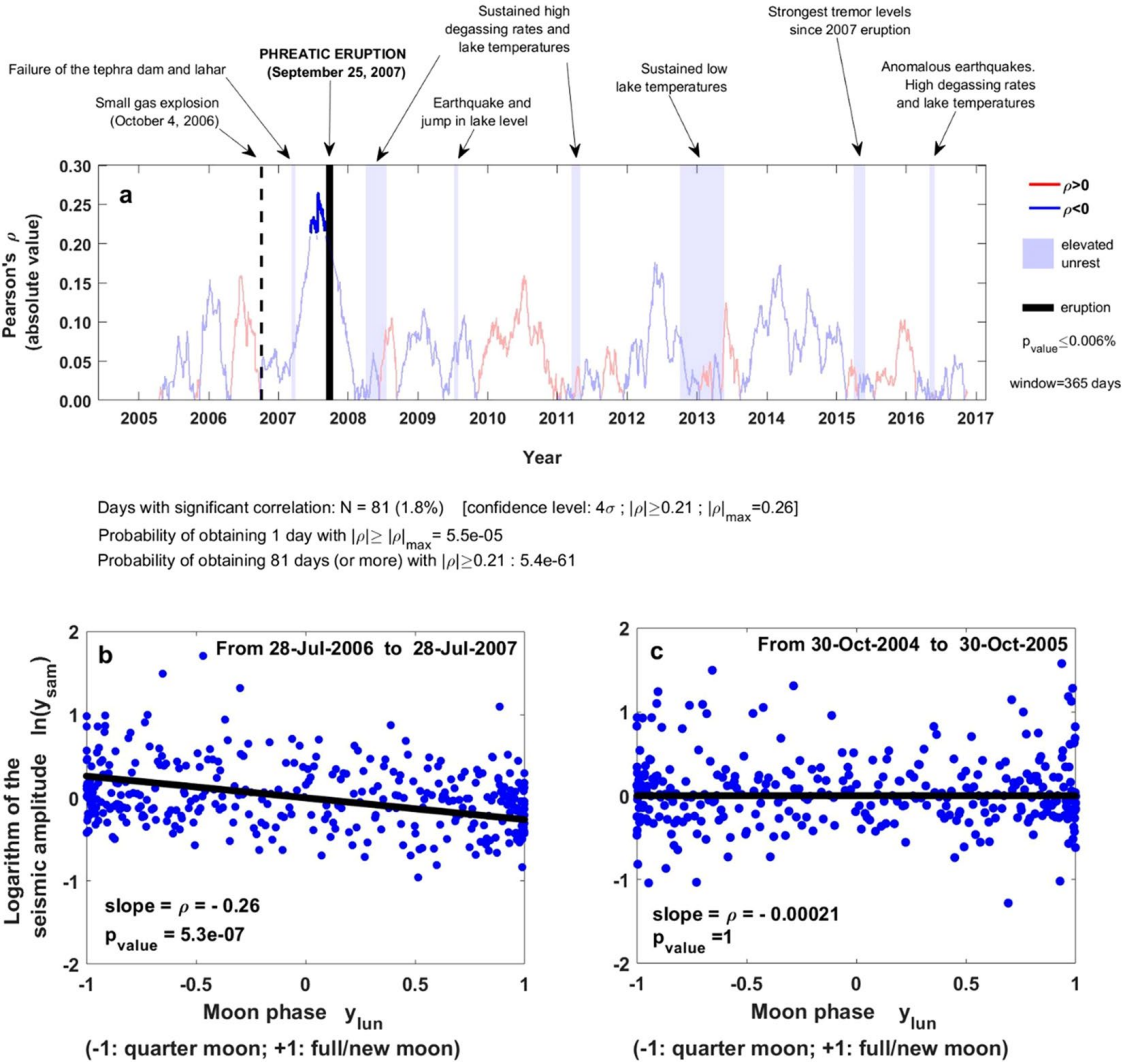
Luni-seismic correlation at Ruapehu. (**a**) Pearson coefficient (*ρ*) for 1-year backward windows. Positive and negative correlations are in red and blue, respectively, shaded regions represent unrest episodes^32^, and deep blue means *p*_*value*_ < 0.006%. This plot depicts the case *N* = 81 days of significant correlation, but we find *N* = 59–86 days (after repeating the method 500 times) depending on the random values used to replace gaps and spikes (Methods, Section 1). The probability of obtaining by chance *N* days with significant correlation is therefore in the range ~10^−36^–10^−64^ (Supplementary Fig. S5). (**b**) Logarithm of the daily median seismic amplitude *ln* (*y*_*sam*_) versus moon phase *y*_*lun*_ (*t*) for the 365 days preceding July 28, 2007; this is the period with maximum correlation obtained. (**c**) *ln* (*y*_*sam*_) versus *y*_*lun*_ (*t*) for the 365 days preceding October 30, 2005; no significant correlation exists in that period.

Our study reveals that the 1-year rolling correlation between lunar cycles and seismic amplitude increases during the ~3 months preceding the phreatic eruption of September 25, 2007 (Fig. 2, Supplementary Fig. S5). During this period, the Pearson coefficient *ρ* is negative (i.e., daily median seismic amplitude tends to be lower towards full/new moon), its magnitude |*ρ*| ≥ 0.21, and it is statistically significant at a confidence level of 4-sigma (i.e., the probability of obtaining the observed *ρ* value -or greater- by chance is lower than 0.006%); the Pearson coefficient reaches a maximum of |*ρ*|_*max*_ = 0.26 with confidence level of 5-sigma about 2 months prior to the eruption (Figs 2a,b). Moreover, we find through Monte Carlo simulations that the probability of obtaining by chance 1 day with |*ρ*| ≥ |*ρ*|_*max*_ is ~10^−5^, whereas the binomial test reveals that the probability of obtaining by chance |*ρ*| ≥ 0.21 over a period extending for ~3 months with a dataset of 12 years is extremely low (<10^−36^). Interestingly, the significant luni-seismic correlation episode arises at a time when Ruapehu did not show signs of unrest, and it disappears after the phreatic eruption. During the rest of the 12-year period analysed, the luni-seismic correlation is not significant beyond the 4-sigma level and the Pearson coefficient *ρ* alternates between positive and negative values that are always lower than ~0.15 in absolute value (Fig. 2a,c). These oscillating low values of *ρ* reveal that the sensitivity of Ruapehu to lunar cycles is independent of the state of unrest, including anomalous episodes with earthquakes, elevated tremor activity, high degassing rates, and abnormally high or low lake temperatures. Our results remain similar if one carries the same analysis with backward windows in the range 290–430 days (Supplementary Fig. S6), so we are statistically confident to claim that Ruapehu was sensitive to lunar cycles during ~9–15 months prior to the 2007 paroxysm. Smaller time windows (<290 days) obscure the luni-seismic correlation prior to the phreatic eruption and lead to some short-term peaks of correlation that emerge sporadically; this is probably due to the instability introduced by the small sample size and the noisy nature of the data^34^. Larger windows (>430 days) also obscure the sensitivity of Ruapehu to lunar cycles; this is so probably because the mechanism behind the seismic response to fortnightly tidal modulation does not operate on so large timescales (Supplementary Fig. S7).

**Figure 3:**
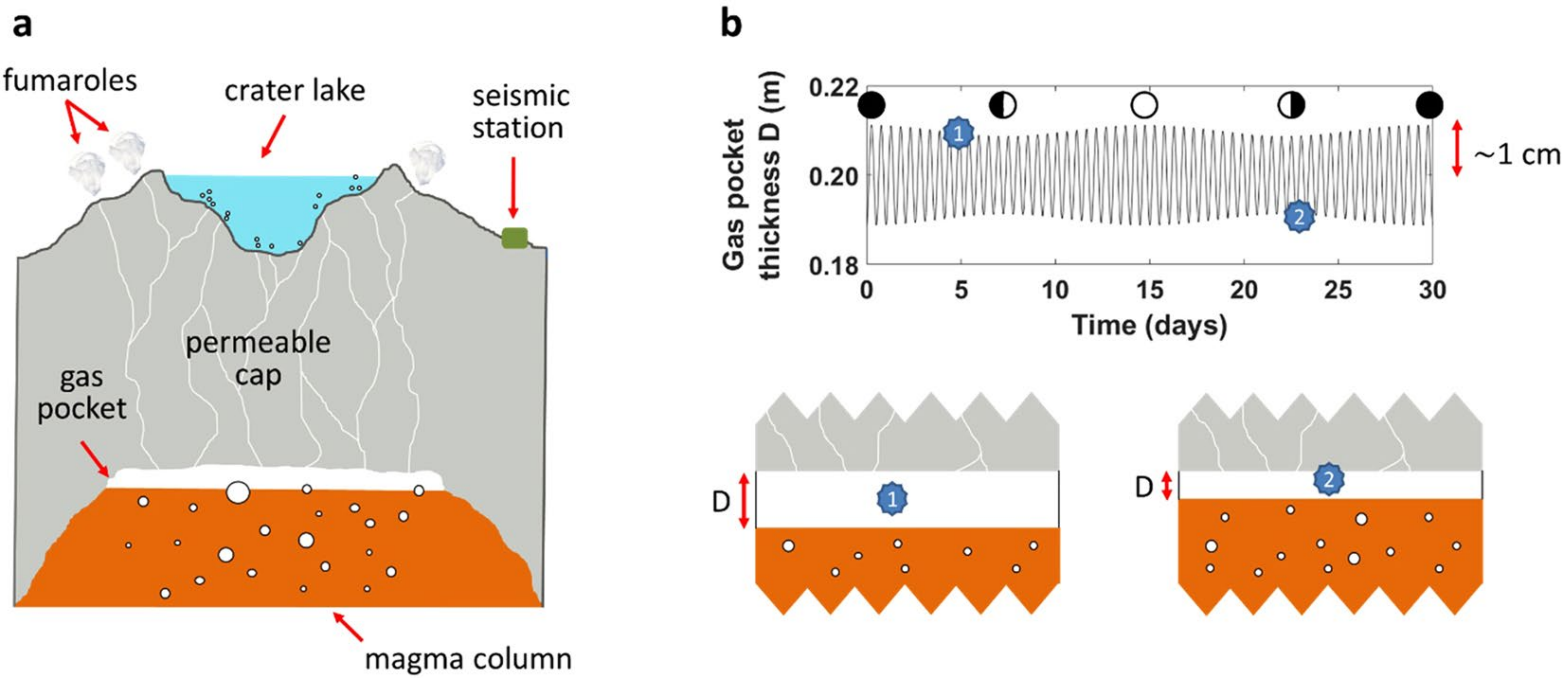
Fundamentals of our model. (**a**) Sketch of Ruapehu volcano during quiescence. Tremor-like pressure oscillations emerge spontaneously in subsurface gas pockets due to the intermittent supply of volatiles into the pocket and the transfer of gases through the porous cap^35,36^. (**b**) Periodic ascent and retreat of magma due to tidal squeezing of the magma plumbing system^1,2,5,6,31^; this generates harmonic variations of the gas pocket thickness (*D*), which in turn affect the amplitude of the seismic signal^35,36^. Black, white, and half disks represent new, full, and quarter moon, respectively. Numbered blue stars represent a case with thick gas pocket and another case with thinner gas pocket.

**Figure 4:**
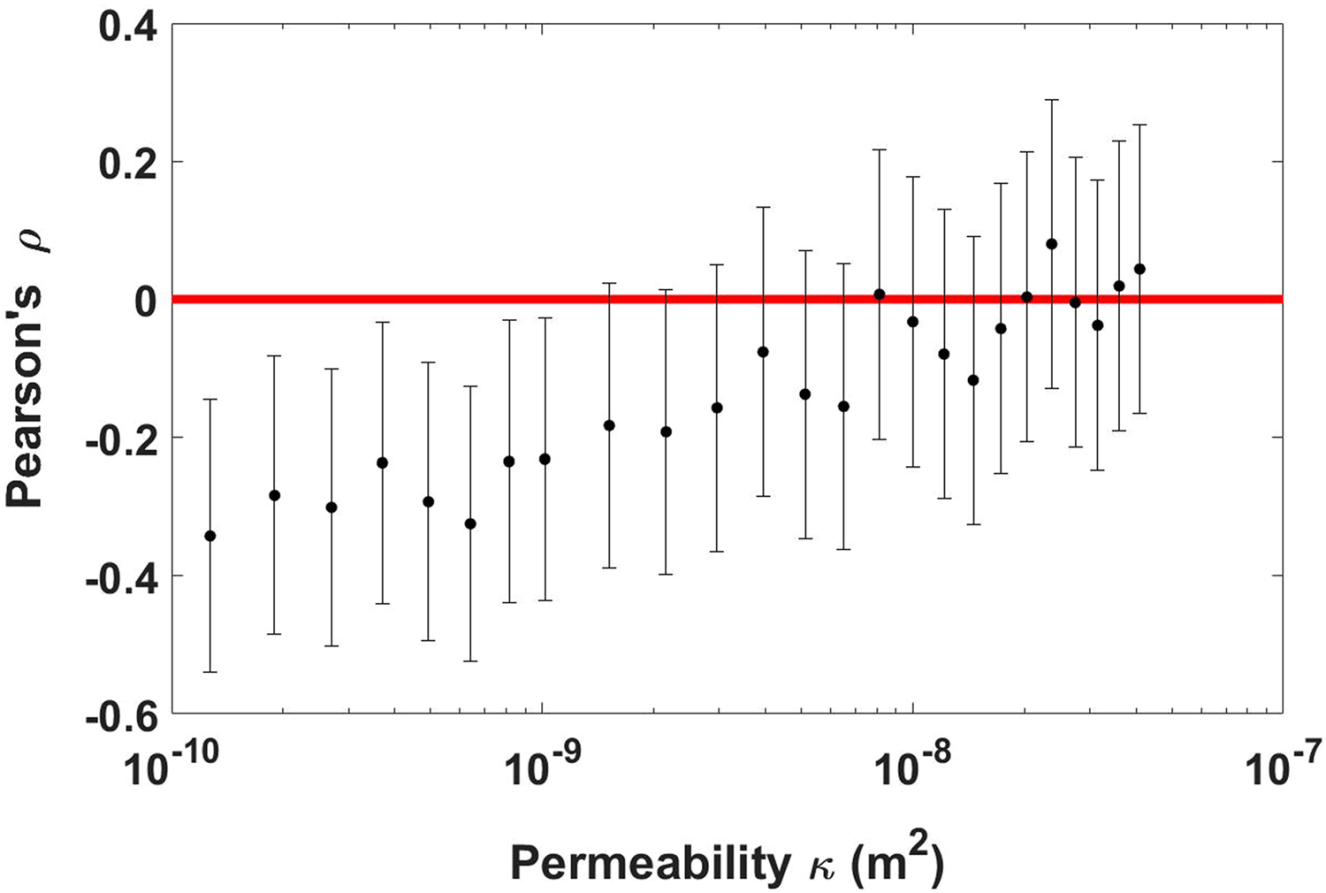
Pearson coefficient (*ρ*) between lunar cycles and the modelled seismic amplitude for different values of the cap permeability (*κ*). Error bars represent four standard deviations of uncertainty (4-sigma confidence level). We use phase shift *δ* = *π* (similar results are obtained with *π*/2 < *δ* < 3*π*/2; see Supplementary Discussion and Supplementary Fig. S13) and realistic values for the different parameters of the model^35,36^ (see details in Methods, Section 3); these values allow generating tremor signals with dominant frequency around 2 Hz (Supplementary Fig. S9), as observed on Ruapehu^37^.

The emergence of long-term (~9–15 months) sensitivity of Ruapehu to fortnightly tides calls for a model to (a) quantify the seismic response of the volcanic system to tidal forcing, and (b) identify the link between luni-seismic correlation and the physical processes that lead to phreatic eruptions (Fig. 3). Based on a recent mechanistic model developed by *Girona et al*.^35,36^, we propose that the persistent seismicity recorded around the crater of Ruapehu (i.e., shallow tremor^37^) is induced by pressure oscillations emerging in gas pockets trapped below the active vent; these pressure oscillations arise in response to two concurrent processes: the permeable flow of gases through the shallowest part of the volcanic edifice and the intermittent supply of volatiles from deeper levels (Fig. 3a). Moreover, we argue that tidal stresses squeeze magma reservoirs, thus inducing harmonic ascent and retreat of magma in the shallow plumbing system^1,2,5,6,31^ (Fig. 3b). Combining the aforementioned approaches, we generate synthetic tremor signals that are analysed in a similar way as the natural data (see details in Methods, Section 3). Our analysis reveals that, for small tidally-induced ascent/retreat of magma (~1 cm amplitude), the magnitude of the luni-seismic correlation increases when the permeability decreases below a threshold value (Fig. 4). In other words, fortnightly tides can modulate the daily median amplitude of volcanic tremor, but only if the permeability of the shallow volcanic edifice is low enough. For example, using realistic values for the different parameters involved in the model, we find that the Pearson coefficient *ρ* differs from zero (at confidence level of 4-sigma) if the permeability *κ* is below ~10^−9^ m^2^, reaching *ρ* = −0.34 ± 0.20 when $\kappa =1.3\cdot {10}^{-10}\,{{\rm{m}}}^{2}$ (note that these values of permeability are realistic for shallow volcanic edifices because they are highly-fractured^38^) (Fig. 4). Our model also suggests that the correlation is negative because the response time^39,40^ of the volcano to tidal stresses is between ~3 h and ~9 h. In turn, this implies that the magma plumbing system of Ruapehu is predominantly under compression when closer to quarter moon and predominantly under extension when closer to full/new moon (see Supplementary Discussion).

We therefore suggest that Ruapehu was sensitive to lunar cycles during 2006/2007 because the shallow vent was highly clogged, which likely triggered the 25 September 2007 phreatic eruption because it favours the pressurization (due to gas over-accumulation) below the active crater^33^. Vent clogging may be induced by two potential mechanisms. First, clogging could occur gradually since the 1995/1996 vent-clearing eruptions due to mineral precipitation in pore networks^33^, thus decreasing the permeability of the vent below threshold values since ~June 2006 (i.e., ~15 months prior to the phreatic eruption). In such a case, gradual clogging maybe also triggered the small 4 October 2006 gas explosion, although no significant luni-seismic correlation could be detected due to the short time window (~90–120 days since June 2006) and the noisy nature of the data^34^. Second, clogging began after the October 2006 gas explosion (~9–11 months prior to the phreatic eruption) due to gravitational compaction and restructuration of pore networks. In such a case, the small gas explosion was probably triggered by other processes not related to vent clogging (e.g., infiltration of meteoric water and subsequent expansion of steam). On the other hand, our model also suggests that Ruapehu is not sensitive to lunar phases since the end of 2007 because the porous vent is building back and its permeability has remained above threshold levels after the phreatic eruption. Hence, the unrest episodes detected during the last 10 years were probably not related to pore clogging of the vent but rather to magma migrations at depth, tectonic processes, or shallower hydrothermal phenomena.

The response of the seismicity recorded around active craters to repeated and predictable tidal excitations has the potential to reveal the state of criticality of the shallowest part of a volcano. In particular, our study reveals that: (a) the persistent seismicity of Ruapehu volcano was not modulated by fortnightly tides over the last 13 years, except for the 9 to 15 months preceding the unpredicted 2007 phreatic eruption; and (b) analysing the correlation between lunar cycles and seismic amplitude offers exciting perspectives to detect the sustained clogging of gas pathways, and thus to better forecast phreatic eruptions at volcanoes whose shallow magma plumbing system is subjected to tidal deformation. Future work should explore whether other volcanoes besides Ruapehu are sensitive to lunar cycles prior to eruptions, although it is worth highlighting that tidal deformation may be more prominent in New Zealand volcanoes than in other volcanoes of the world. This is so because ocean-tide loading is larger in New Zealand than in most other places, and its location in mid-latitudes implies that Earth tides are also strong^41,42^. The only requirement to apply our statistical approach to other volcanoes is to have mostly continuous and long-lasting (several years) seismic data recorded nearby volcano craters. Our interpretation of the luni-seismic correlation is applicable to any volcano with prominent outgassing during quiescence, and thus when tremor is controlled by the permeable flow of gases through the shallowest part of the volcanic edifice^35,36^.

## Methods

### Data Processing

#### Data processing consists of the following steps

- Seismic data are obtained from a permanent short-period seismic station installed on top of Ruapehu volcano (~700 m from the crater lake). In particular, we use the vertical component waveform over a period spanning from February 22, 2004, to November 15, 2016 (downloaded from the open access Geonet-GNS archive, ref.^32^). This time period was selected because the same type of seismometer was consistently used; we note that the seismometer was destroyed during the 2007 eruption, although it was replaced a few days later by the same type of sensor.
- The daily median seismic amplitude *y*_*sam*_ (*t*) is computed through the following standard procedure: we read files containing velocity data over a duration of 1 day, subtract the mean of the 1-day time series, apply a high-pass filter (0.1 Hz, butterworth, 4 corners), integrate to obtain the displacement waveform, apply the same high-pass filter (0.1 Hz) followed by a low-pass filter (40 Hz, butterworth, 4 corners), take the absolute value of the displacement waveform, calculate the median of the absolute values in every window of 90 s (our results are essentially the same when using window durations ranging between 30 s and 1800 s; Supplementary Fig. S8), and export the median of all the windows contained in each day (960 windows when using window durations of 90 s). This procedure, which is repeated for every day of the 12-year period, allows capturing the persistent seismicity of Ruapehu (i.e., tremor^37^) by minimizing the potential contamination due to meteorological perturbations, tectonic earthquakes, or other unwanted non-volcanic effects. For our analysis, we finally calculate the logarithm of the daily median seismic amplitude *ln*(*y*_*sam*_).
- Gaps (e.g., after the phreatic eruption of 2007, which destroyed the seismic station) and spikes (probably due to storms or electronic problems), which only represent ~5% of the *ln*(*y*_*sam*_) time series, are replaced by random values obtained from a normal distribution with the same mean and standard deviation as the data. The replacement of gaps and spikes allows us to apply a 20-day high-pass median filter to remove variations of the seismic amplitude occurring in timescales larger than the average periodicity of high tides (Supplementary Fig. S1).

### Data Analysis

The correlation between lunar cycles and seismic amplitude (defined here as luni-seismic correlation) is analysed as follows:

- We create a synthetic periodic time series to describe lunar phases (Supplementary Fig. S2): *y*_*lun*_ = −*cos*(2*π*(*t* − *t*_*low*_)/*T*), where *t* is an integer representing time in days, *t*_*low*_ is a reference day of the calendar with low tide (i.e., quarter moon), and *T* = 14.7653 days is the average periodicity of high or low tides (i.e., the average time between a full moon and the next new moon). With this approach, *y*_*lun*_ = 1 during high tides and *y*_*lun*_ = −1 during low tides. We choose February 28, 2004, as our reference day with low tide (*t*_*low*_). Note that we produce a value of *y*_*lun*_ per day; the time of the day at which *y*_*lun*_ is generated is the same as the peak of quarter moon on the reference day.
- We calculate the Pearson correlation coefficient (*ρ*) with different subsets of the time series *ln*(*y*_*sam*_) and *y*_*lun*_ (Supplementary Fig. S3). These subsets are backward windows of *L* days, such that the value of *ρ* corresponding to a given day is calculated with the *L* data pairs preceding that day. It is worth noting that: I) *ρ* evaluates how well two datasets are linearly correlated, such that 0 < *ρ* ≤ 1 implies a positive correlation (i.e., *ln*(*y*_*sam*_) tends to increase with *y*_*lun*_) and −1 ≤ *ρ* < 0 implies a negative correlation (i.e., *ln*(*y*_*sam*_) tends to decrease with *y*_*lun*_). II) The subsets of *ln*(*y*_*sam*_) and *y*_*lun*_ are standardized, i.e., the mean of each time series is subtracted and the result is divided by the standard deviation. This standardization process does not affect the value of *ρ* and makes it match with the slope of the line that best fits *ln*(*y*_*sam*_) and *y*_*lun*_(*t*) (Fig. 2b,c). III) The random values previously added to replace gaps and spikes are not taken into account to calculate *ρ*. IV) *ρ* is calculated for backward windows whose number of gaps and spikes is less than 20% of the number of days *L*. V) Larger values of *L* allow analyzing sustained long-term correlations while ensuring the applicability of statistical methods^34^. In our analysis we focus on *L* = 1 year, although the results are similar for backward window sizes in the range 290–430 days (Supplementary Fig. S6).
- We test whether *ρ* obtained for every moving window is significantly different from zero. To do this, we perform a significance test consisting of calculating the probability (*p*_*value*_) of obtaining the observed correlation coefficient (*ρ*) if the data pairs of a given window are not correlated (null hypothesis); the probability *p*_*value*_ is calculated with a two-tailed test (MATLAB algorithm). If *p*_*value*_ ≤ *P*, where *P* is a given threshold value, the correlation coefficient *ρ* is said to be statistically significant at a given confidence level. For example, if *p*_*value*_ ≤ 0.006% for a given window, the correlation between *ln*(*y*_*sam*_) and *y*_*lun*_ in that window is different from zero at a confidence level of 4-sigma. In other words, the probability that the seismic amplitude and lunar cycles can be correlated by chance in that window is lower than 0.006%. Here, we use 4-sigma as confidence level to reject or not the null hypothesis, a much stricter condition than has been considered so far in other studies focusing on the influence of moon cycles on volcanic activity (usually 2-sigma or lower^3,20,25^).
- We test the extent to which a stochastic seismic amplitude time series, which is known to be unrelated with lunar cycles, may give rise to the significant correlation coefficients (*ρ*) observed in the natural data. In other words, we quantify whether the values and duration of significant correlation obtained (with confidence 4-sigma) can be produced with a random seismic amplitude time series. This is done by following the next steps: I) we compute the number of days *N* that satisfies |*ρ*| ≥ |*ρ*_*sig*_| with the natural data, where |*ρ*_*sig*_| is the minimum correlation coefficient (in absolute value) from which the level of confidence is 4-sigma. II) We build a random seismic amplitude time series $ln({y}_{sam}^{rand})$ with the same mean and standard deviation as the natural data *ln*(*y*_*sam*_). III) We calculate the Pearson correlation coefficient between $ln({y}_{sam}^{rand})$ and the moon phase time series *y*_*lun*_(*t*), exactly as we did for the natural data (i.e., for moving windows of size *L* and assuming the same gaps and spikes). IV) We repeat the previous step 500 times (Monte Carlo simulations) to obtain the probability density function of correlation coefficients. For example, for *L* = 1 year, we obtain a Gaussian probability density function with mean equal to 0 and standard deviation equal to 0.0659 (Supplementary Fig. S4). V) Using the aforementioned probability density function, we calculate the probability of obtaining by chance one day with correlation coefficient that is equal or larger than the minimum significant correlation obtained with the data (in absolute value; |*ρ*_*sig*_|). VI) We use the previous result to perform a binomial test; this test allows us to calculate the probability of obtaining, in the more than 12 years of data, *N* days (or more) with correlation coefficient satisfying |*ρ*| ≥ |*ρ*_*sig*_|.

### Model

We propose a mechanical model for tremor based on *Girona et al*.^35,36^. We use this forward model to generate synthetic seismic datasets and study the factors that can cause a change in the correlation between lunar cycles and the observed seismicity.

#### Mechanical model of shallow tremor

We propose that shallow tremor at Ruapehu arises from pressure oscillations Δ*P* occurring in a shallow gas pocket embedded beneath the volcanic crater (called steam zone by other authors^37^). These pressure changes Δ*P* do not result from elastic oscillations of the shallow conduit, but emerge in response to two concurrent processes: the permeable flow of gases through the shallow cap and the intermittent supply of volatiles from deeper levels. To first order, Δ*P* beneath volcanic craters is given, in the frequency domain, by^35,36^:

1 $${\rm{\Delta }}P(\omega )=\frac{{R}_{g}{T}_{g}}{\pi {R}^{2}DM}\{-\frac{4{\Gamma }_{1}{Q}_{0}\pi }{{\omega }_{0}^{2}}\delta (\omega )+\frac{({\Gamma }_{1}+j\omega )(1+{e}^{-j\omega {t}_{s}})}{{\omega }_{0}^{2}-{\omega }^{2}(1+{e}^{-j\omega {t}_{s}})+j\omega (\Gamma +{\Gamma }_{1}{e}^{-j\omega {t}_{s}})}\sum _{n=1}^{{N}_{\ast }}{q}_{n}{e}^{-j\omega {t}_{n}}\},$$

where *R*_*g*_ is the ideal gas constant, *T*_*g*_ is the gas temperature, *R* is the radius of the uppermost part of the volcanic conduit, *D* is the thickness of the gas pocket embedded beneath the crater, *M* is the molecular weight of water (H_2_O is the main component of volcanic gas emissions), *Q*_0_ is the mean outgassing flux, *δ*(*ω*) is the Dirac delta function (and thus *δ*(*ω*) > 0 for *ω* > 0), *t*_*s*_ is the seepage time (i.e., time required for the gas to pass through the permeable cap), *q*_*n*_ is the mass of gas introduced into the gas pocket at the instant *t*_*n*_ (e.g., through bubble bursting at the top of a fluid-like magma column), N_*_ is the total number of mass impulses (e.g., number of bubbles that burst at the top of the magma column) occurring in the gas pocket during the simulation time, *ω* is the angular frequency, and *j* is the imaginary unit. The parameters *Γ*_1_, *Γ*, and ${\omega }_{0}^{2}$ are defined as *Γ*_1_ = 2*μR*_*g*_*T*_*g*_*φ*/[(*P*_0_ + *P*_*ex*_*)Mκ*], *Γ* = *Γ*_1_ + 2*R*_*g*_*T*_*g*_*Q*_0_/[(*P*_0_ + *P*_*ex*_)*πR*^2^*DM*], and ${\omega }_{0}^{2}=4{P}_{0}{R}_{g}{T}_{g}\phi /[({P}_{0}+{P}_{ex})DM{L}_{c}]$, where *μ* is the gas viscosity, *P*_0_ is the mean pressure in the gas pocket, *P*_*ex*_ is the pressure at the exit vent (i.e., hydrostatic pressure at the bottom of the crater lake), *L*_*c*_ is the cap thickness, and *φ* and *κ* are the connected porosity and permeability of the cap, respectively. In turn,the mean pressure in the gas pocket (*P*_0_) and the seepage time (*t*_*s*_) can be calculated from:

2 $${P}_{0}={P}_{ex}\sqrt{1+\frac{2{L}_{c}\mu {R}_{g}{T}_{g}{Q}_{0}}{\kappa \pi {R}^{2}M{P}_{ex}^{2}}}$$

and

3 $${t}_{s}=\frac{({P}_{0}+{P}_{ex})M\phi \pi {R}^{2}\tau {L}_{c}}{2{Q}_{0}{R}_{g}{T}_{g}},$$

where *τ* is the tortuosity (i.e., ratio of the actual path length for the gas to escape through the cap to the cap thickness) and the other parameters have been previously defined. The parameters *φ* and *κ* are assumed to be related through the following empirical function:

4 $$k={k}_{\ast }{(\frac{\phi }{{\phi }_{\ast }})}^{\alpha },$$

where φ_*_ and κ_*_ are two constants, and the exponent *α* ranges between 1 and 25 for common geologic materials (we use here: ${\phi }_{\ast }=3\cdot {10}^{-4}$, κ_*_ = 10^−8^ m^2^, and *α* = 3; see refs^35,36^ for details).

By convolving equation (1) with the Green’s function describing the propagation of Rayleigh waves along the path to the receiver, we can compute the vertical ground displacement that would be recorded at nearby stations. The vertical component of the ground displacement *u*_*z*_ is given, in the frequency domain, by^35,36^:

5 $$\begin{array}{rcl}{u}_{z}(\omega ) & = & [\frac{{R}_{g}{T}_{g}}{DM}\frac{({{\Gamma }}_{1}+j\omega )(1+{e}^{-j\omega {t}_{s}})}{{\omega }_{0}^{2}-{\omega }^{2}(1+{e}^{-j\omega {t}_{s}})+j\omega (\Gamma +{\Gamma }_{1}{e}^{-j\omega {t}_{s}})}]\,[\sum _{n=1}^{{N}_{\ast }}{q}_{n}{e}^{-j\omega {t}_{n}}]\\  &  & \times [\sqrt{\frac{2{v}_{c}\omega }{\pi r}}\frac{{e}^{-\frac{\omega r}{2{v}_{u}{Q}_{f}}}}{8{\rho }_{s}{v}_{c}^{2}{v}_{u}}\,{e}^{j(\frac{\omega r}{{v}_{c}}+\frac{\pi }{4})}],\end{array}$$

where *v*_*c*_ is the phase velocity (we use *v*_*c*_ = 1295(*ω*/2*π*)^−0.374^ m/s), *r* is the distance from the source to the receiver, *v*_*u*_ is the group velocity (we use *v*_*u*_ = 0.73*v*_*c*_), *Q*_*f*_ is the dimensionless quality factor (a parameter that accounts for the attenuation of seismic waves), and *ρ*_*s*_ is the density of the medium. The parameters *v*_*c*_, *v*_*u*_, *Q*_*f*_, and *ρ*_*s*_ are related to the propagation of the seismic waves through the crust and not to the seismic source. The ground displacement described by equation (5) was shown to explain the main features of shallow volcanic seismicity, particularly monochromatic tremor as typically recorded around Ruapehu (Supplementary Fig. S9).

#### Effect of tidal stresses on the parameters of the model

The continuous compressions-extensions induced by tidal stresses in the shallow crust are thought to squeeze magma reservoirs^1,2,5,6,31^. This, in turn, is expected to induce harmonic ascent and retreat of magma in shallow plumbing systems (as sometimes observed in Kilauea^1,2,5,6^ and Villarrica lava lakes^31^), thus changing the thickness of the gas pocket (*D*) with time. The effect of tidal stresses on the gas pocket thickness is parameterized with a sum of harmonic time series:

6 $$D(t)={D}_{0}+\sum _{i}{D}_{i}\,{\cos }(2\pi t/{T}_{i}+\delta ),$$

where *D*_0_ is the mean gas pocket thickness, the parameters *T*_*i*_ and *D*_*i*_ represent the period and amplitude, respectively, of each tidal constituent *i*, and *δ* is a phase shift that accounts for the response time of the volcano to tidal stresses. In particular, *δ* is the phase of the daily oscillation of the gas pocket thickness at a specific time *t* and moon phase, which depends on how the combination of Earth tides and ocean-tide loading in New Zealand deforms the magma plumbing system of Ruapehu (see Supplementary Discussion). For simplicity, we consider only the tidal constituents responsible for generating fortnightly tidal modulation, i.e., the principal lunar semidiurnal (*M*_2_), with periodicity ${T}_{{M}_{2}}=12.42\,\,$hours; and the principal solar semidiurnal (*S*_2_), with periodicity ${T}_{{S}_{2}}=12\,\,$hours. We also assume tidal strains to be small and use ${D}_{{M}_{2}}=0.01$ m and ${D}_{{S}_{2}}={D}_{{M}_{2}}/8$. With these values, the gas pocket thickness *D*(*t*) reproduces the classic fortnightly spring/neap cycle (Fig. 3b), with maximum amplitude of the fluctuations on the order of 0.01 m. This amplitude is considered a minimum end-member; tidally-induced magma level oscillations of up to 30–60 cm were observed sometimes at Halemaumau lava lake^1,2,5,6^. Finally, we explore values of the phase shift in the range *δ* = 0 − 2*π*. The simple approach used in this study allows us to analyse how small harmonic variations of the gas pocket thickness due to tidal stresses affect the amplitude of the synthetic seismic time series. It is worth noting that, as revealed by equation (6), we also expect semi-diurnal modulation of the seismic amplitude (Supplementary Fig. S10); this has been observed at Fogo volcano, Cape Verde Republic^42^, and can be sometimes detected by simple eye inspection at Ruapehu (Supplementary Fig. S11).

#### Synthetic data processing

To analyse the correlation between lunar cycles and the logarithm of the modelled daily seismic amplitude, we proceed as follows:

- We assign the following realistic values to the parameters of the model: external pressure ${P}_{ex}=2.06\cdot {10}^{6}$ Pa (i.e., atmospheric pressure + hydrostatic pressure of a 200 m water lake, consistent with historical lake depths at Ruapehu^43^), cap thickness *L*_*c*_ = 10 m (this implies that tremor is sourced beneath the crater lake at a depth consistent with previous studies^44^), radius of the shallow magma conduit and cap *R* = 10 m, mean thickness of the gas pocket *D*_0_ = 0.1 m, mean outgassing flux *Q*_0_ = 50 kg/s, volcanic gas temperature *T*_*g*_ = 900 °C, gas viscosity *μ* = 10^−5^ Pas, molecular weight of gas (mostly water vapour) *M* = 0.018kg/mol, cap porosity *φ* = *φ*_0_(*κ*/*κ*_0_)^1/*α*^, with ${\phi }_{0}=3\cdot {10}^{-4}$, *κ*_0_ = 10^−8^ m^2^, and *α* = 3 (realistic for highly fractured caps and permeable flow dominated by open cracks and channels^35,36^), and tortuosity *τ* ≈ 1. Besides, we impose the random supply of N_*_ = 1,000 gas bubbles in 90 s of simulation, and we use distance source-receiver *r* = 700 m, density of the medium *ρ*_*s*_ = 3,000 kg/m^3^, frequency-dependent phase velocity *v*_*c*_ = 1295(*ω*/2*π*)^−0.374^ m/s, group velocity *v*_*u*_ = 0.73*v*_*c*_, and dimensionless quality factor *Q*_*f*_ = 5. These values of the parameters generate mean pressures in the gas pocket *P*_0_ on the order of 10^6^ Pa (slightly greater than the external pressure *P*_*ex*_) and tremor signals with dominant frequency around 2 Hz (Supplementary Fig. S9), as observed on Ruapehu^37^.
- For the given mean gas pocket thickness *D*_0_ (and for a given value for the phase shift *δ*), we calculate *D* at *t* = 0 using equation (6). Then, we simulate a 90 s time series of ground displacement *u*_*z*_(t) by calculating the inverse Fourier transform of equation (5) (details on the calculation in ref.^35^). This mimics the time windows that were used in the analysis of the natural data (see 1. Data Processing). Later, we calculate the seismic amplitude of the 90 s simulation by taking the absolute value of the synthetic displacement waveform and computing the median. Note that the gas pocket thickness *D* is assumed to remain constant during the 90 s simulation because it varies over longer timescales.
- We repeat the aforementioned calculations after recalculating the gas pocket thickness *D* over steps of 15 minutes for a duration of *t* = 1 year (the time step is limited to 15 minutes to make the problem more tractable in terms of computer run time). This gives a total of 96 seismic amplitudes per day (instead of the 960 values per day that we have with the data), from which we take the median to obtain the daily seismic amplitude and take its logarithm. We also create a synthetic periodic time series to describe lunar cycles, exactly as we did to analyse the natural data (see 2. Data Analysis).
- The procedure described above allows us to generate model data that we can treat statistically to test the conditions that are required to explain an increase of luni-seismic correlation prior to the 2007 phreatic eruption of Ruapehu. In particular, we conduct the same statistical analysis as with the natural data, i.e., we calculate the Pearson correlation coefficient *ρ* between the logarithm of the modelled daily seismic amplitude and the lunar cycles (Supplementary Fig. S12). This is done for 365 data pairs (1 year of synthetic data).
- Finally, we repeat steps b–d with different values of the cap permeability *κ* and porosity *φ* (related through equation (4)) to explore how overall cap sealing (e.g., due to pore mineralization or subsidence of the crater floor after the 2006 gas explosion) affects the luni-seismic correlation. This analysis reveals that the luni-seismic correlation is significant when the permeability of the cap *κ* is below a threshold value; and it is negative as long as the phase shift *δ* meets *π*/2 < *δ* < 3*π*/2 and hence if the response time of the volcano to tidal stresses ranges between ~3 h and ~9 h (see Supplementary Discussion and Supplementary Fig. S13).

## Electronic supplementary material

Supplementary Material
