# Supplementary material for: Sensitivity to lunar cycles prior to the 2007 eruption of Ruapehu volcano
Source: Sci Rep. 2018 Jan 24;8:1476. doi: 10.1038/s41598-018-19307-z (PMC5784125; doi:10.1038/s41598-018-19307-z)
Supplement: Supplementary file 1 — Supplementary Material [file 41598_2018_19307_MOESM1_ESM.pdf]

# Sensitivity to lunar cycles prior to the 2007 eruption of Ruapehu volcano

Társilo Girona[1,2*], Christian Huber[1], Corentin Caudron[3,4,5]

[1]Department of Earth, Environmental and Planetary Sciences, Brown University, Providence, Rhode Island, USA.

[2]Jet Propulsion Laboratory, California Institute of Technology, Pasadena, CA 91109, USA (current affiliation).

[3]Département Géosciences, Environnement et Société, Université Libre de Bruxelles (ULB), Belgium.

[4]Department of Seismology and Gravimetry, Royal Observatory of Belgium, Uccle, Belgium.

[5]Department of Geology, Ghent University, Krijgslaan 281 (S8-WE13), Ghent, Belgium (current affiliation).

*Correspondence to: tarsilo.girona@jpl.nasa.gov.

**Description.** Supplementary Discussion and Supplementary Figures

**Supplementary Discussion**

For the given set of geometrical and physical parameters used to model shallow tremor at Ruapehu volcano, we find that the significant luni-seismic correlation is positive (i.e., daily median amplitude of tremor is higher at the full/new moon) or negative (i.e., daily median amplitude of tremor is higher at the quarter moon) depending on the phase shift $\delta$ appearing in equation (6) of the manuscript. To illustrate the physical meaning of $\delta$ and thus to interpret the sign of the correlation, we focus first on how the time series describing lunar cycles, the gas pocket thickness $D(t)$, and the synthetic seismic amplitude vary along one day with full/new moon and $\delta = \pi$. It is met that:

- The time series describing lunar cycles varies very little because the average periodicity of high or low tides is much larger than one day (~14.8 days).

- The gas pocket thickness shows daily oscillations that result from the superposition of the dominant tidal constituents (i.e., principal lunar semidiurnal, with periodicity 12.42 h; and principal solar semidiurnal, with periodicity 12 h) (Supplementary Fig. S10). These oscillations result from the daily variations of tidal compression in the magma plumbing system; lower values of the gas pocket thickness means that magma is higher up in the conduit because the plumbing system is tidally compressed, whereas larger values of the gas pocket thickness means that magma is lower down in the conduit because the plumbing system is under extension regime.

- The seismic amplitude responds to the daily oscillations of the gas pocket thickness (Supplementary Fig. S10). In particular, the lower the porosity and permeability of the cap, the more sensitive the seismic amplitude to the variations of the gas pocket thickness. For very low

porosity and permeability, seismic amplitude increases systematically when the gas pocket thickness decreases.

From the 96 values of seismic amplitude calculated along one day (one every 15 minutes to make the problem tractable in term of computer run time), we calculate the daily median value that is then used in our statistical analysis. For example, for the day analysed in Supplementary Fig. S10, the gas pocket thickness is most of the time larger than the mean thickness $D_0$ and thus the median amplitude of tremor reaches values that are lower than the long-term (>15 days) average. This means that, for phase shift $\delta = \pi$, extension predominates over compression around high tides and thus daily median amplitude tends to be lower close to full/new moon. By repeating our numerical simulations with different values of the phase shift $\delta$, we find that (Supplementary Fig. S13):

(a) If $\frac{\pi}{2} < \delta < \frac{3\pi}{2}$ → Extension predominates over the days around full/new moon and compression predominates over the days around quarter moon. In this case, negative correlation emerges between daily median amplitude of tremor and lunar cycles when the permeability of the cap is below a threshold value.

(b) If $-\frac{\pi}{2} < \delta < \frac{\pi}{2}$ → Compression predominates over the days around full/new moon and extension predominates over the days around quarter moon. In this case, positive correlation emerges between daily median amplitude of tremor and lunar cycles when the permeability of the cap is below a threshold value.

(c) If $\delta \sim \pm\pi/2$ → Neither compression nor extension predominates and thus no difference

exists between high tides and low tides. In this case, the correlation is always very weak independently of the permeability of the cap.

Based on these results, we conclude that the condition $\frac{\pi}{2} < \delta < \frac{3\pi}{2}$ applies to Ruapehu volcano. On the other hand, each harmonic component of equation (6) can be written as:

$$\cos\left(\frac{2\pi t}{T_i} + \delta\right) = \cos\left(\frac{2\pi t}{T_i} + \frac{\delta T_i}{T_i}\right) = \cos\left(\frac{2\pi(t + \delta T_i/2\pi)}{T_i}\right) = \cos\left(\frac{2\pi(t + \tau_r)}{T_i}\right) \quad , \quad (S1)$$

where $\tau_r = \delta T_i/2\pi$ can be interpreted as the response time of the magma plumbing system to tidal stresses[38,39]. Taking into account that the condition $\frac{\pi}{2} < \delta < \frac{3\pi}{2}$ applies to Ruapehu volcano and that the periodicity of the tidal constituents is $\sim$12 h, we find that the response time $\tau_r$ meets:

$$3\text{ h} < \tau_r < 9\text{ h} \quad . \qquad (S2)$$

Hence, the luni-seismic correlation that emerges prior to the 2007 eruption of Ruapehu is negative because the response time of the volcanic system to tidal stresses is in the range $\tau_r \sim 3\text{ h} - 9\text{ h}$; in turn, this implies that tidal compression predominates over the days around quarter moon. It is worth stressing that this does not mean that compression only exists over quarter moons (cycles of compression-extension occurs daily), but that the magma plumbing system remains more hours under compression than under extension when closer to quarter moon.

## Supplementary Figures

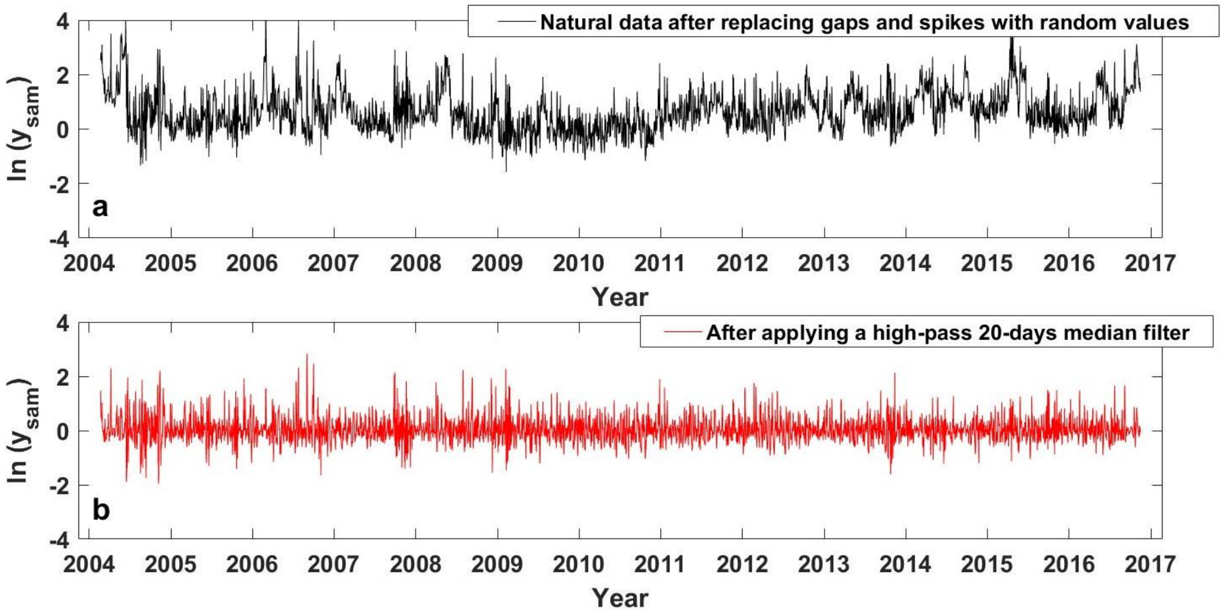


**Supplementary Figure S1. Variation of the seismic amplitude $(y_{sam})$ with time. a,** Natural data after replacing gaps and spikes with random values. **b,** Natural data after replacing gaps and spikes with random values, and applying a high-pass 20-days median filter.

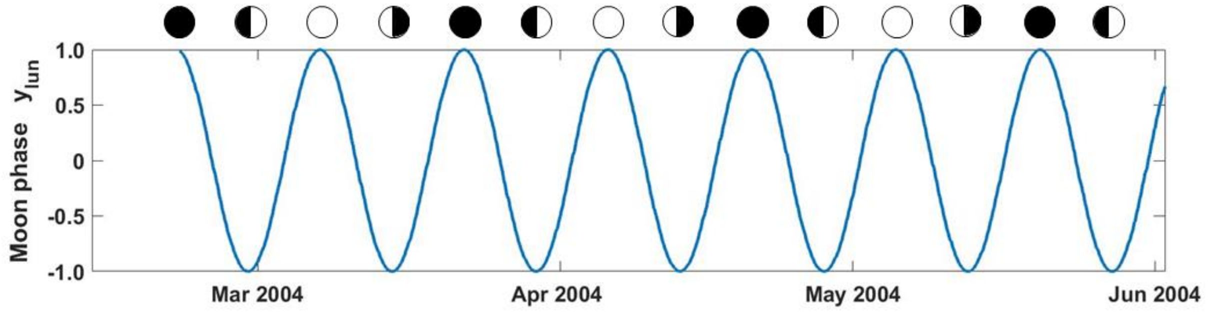


**Supplementary Figure S2. Synthetic time series describing lunar phases ($\boldsymbol{y_{lun}}$).** It is met that $y_{lun} = 1$ during high tides (full moon -white circle- or new moon -black circle-) and $y_{lun} = -1$ during low tides (quarter moon).

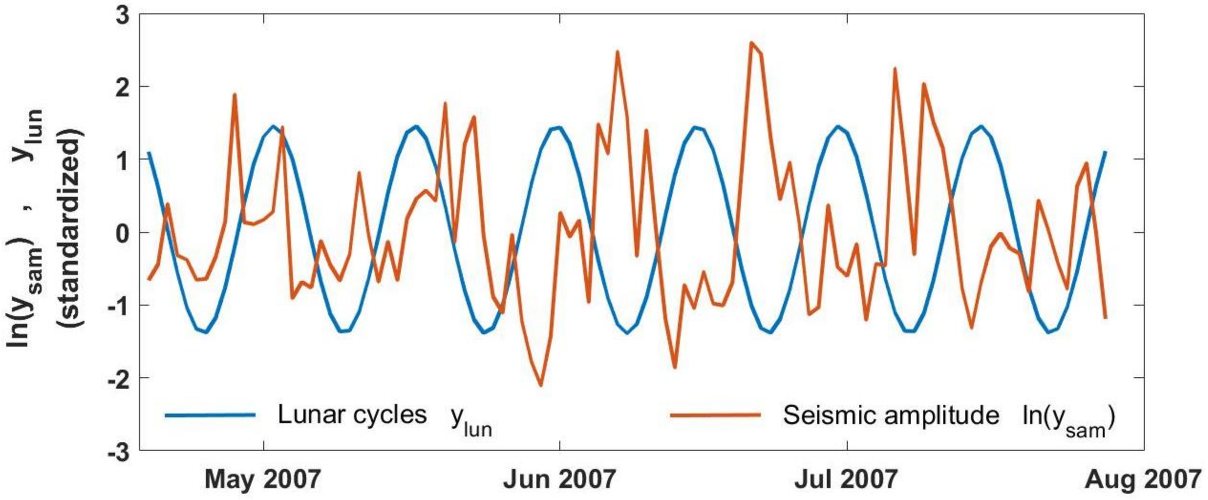


**Supplementary Figure S3. Comparison of the seismic amplitude time series and moon phase in a 4-months period before the 2007 phreatic eruption.** Both time series are standardized (i.e., the mean is removed and the result is divided by the standard deviation).

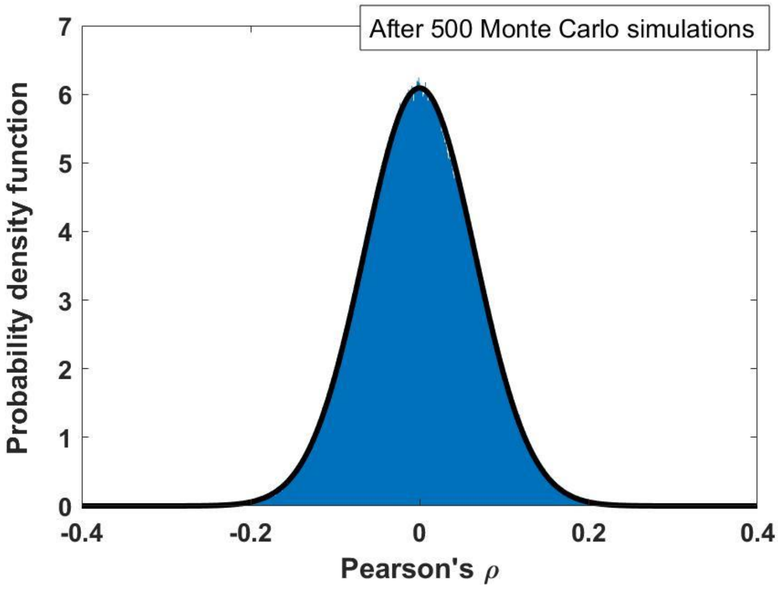


**Supplementary Figure S4. Probability density function of the Pearson correlation coefficient ($\rho$).** This is obtained by calculating the correlation coefficients between the moon phase time series and 500 random seismic amplitude time series with the same mean and standard deviation as the natural data ($ln(y_{sam})$). The distribution of $\rho$ is Gaussian, with mean equal to 0 and standard deviation equal to 0.0659 (for moving windows of $L = 1$ year). We assume the same gaps and spikes as in the natural data.

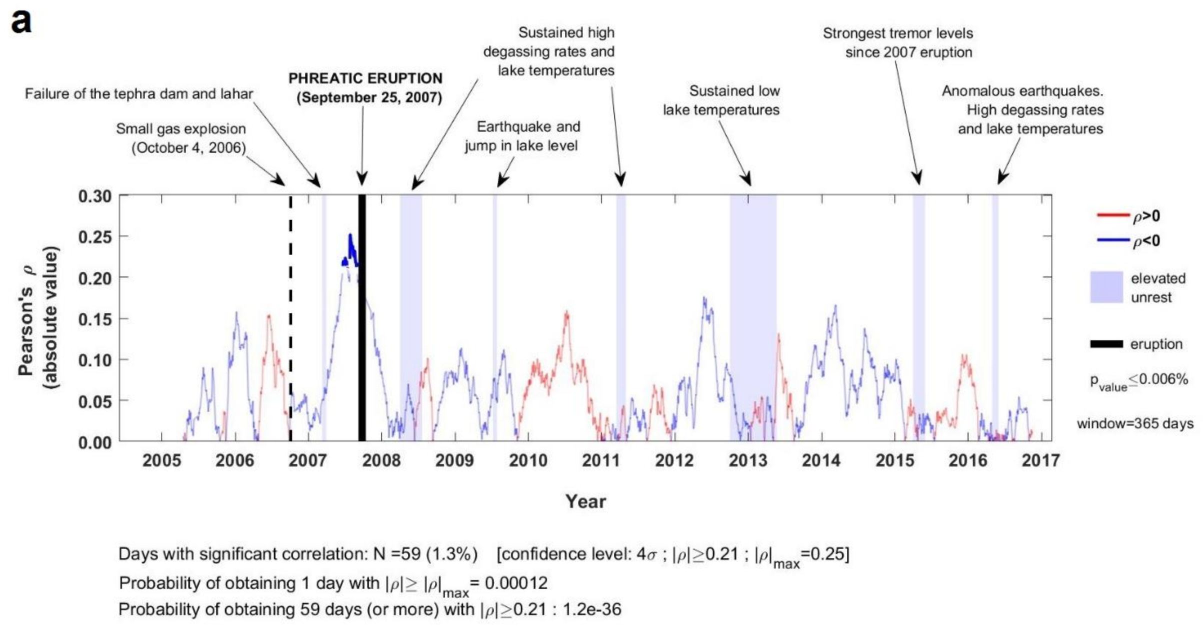


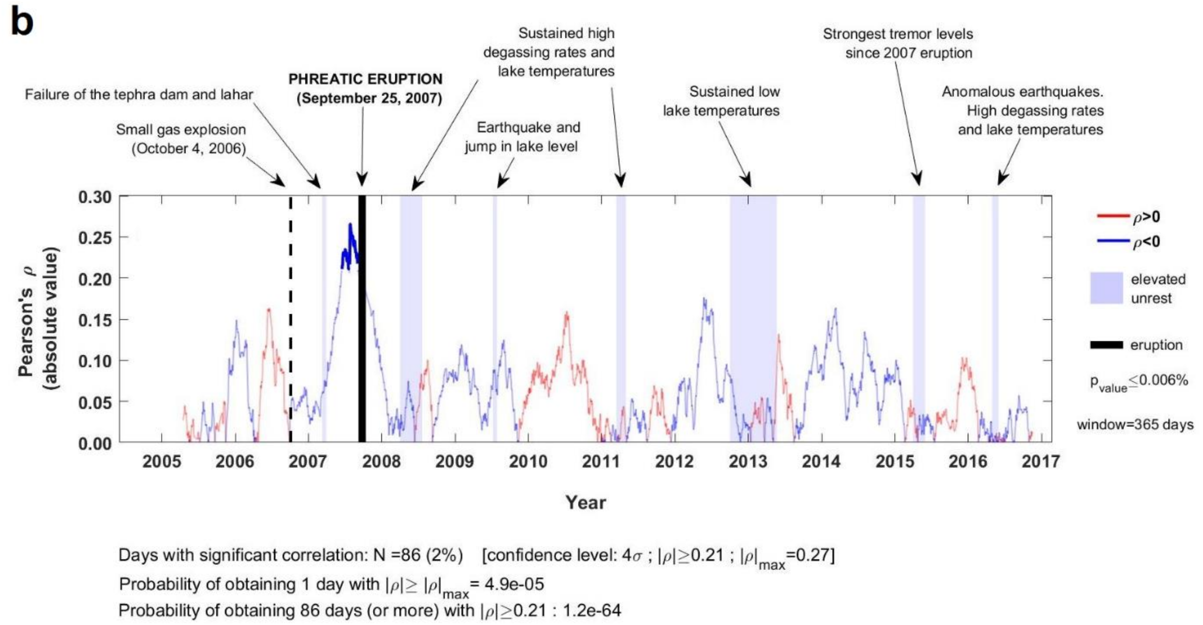


**Supplementary Figure S5. End-members of luni-seismic correlation obtained for Ruapehu volcano after repeating our analysis 500 times. a,** Correlation time series with the minimum number of days with significant correlation (N=59 days). **b,** Correlation time series with the maximum number of days with significant correlation (N=86 days). Different values are obtained as result of the random values used to replace gaps and spikes (see Methods, Section 1). See caption of Figure 2 for further explanations of the panels.

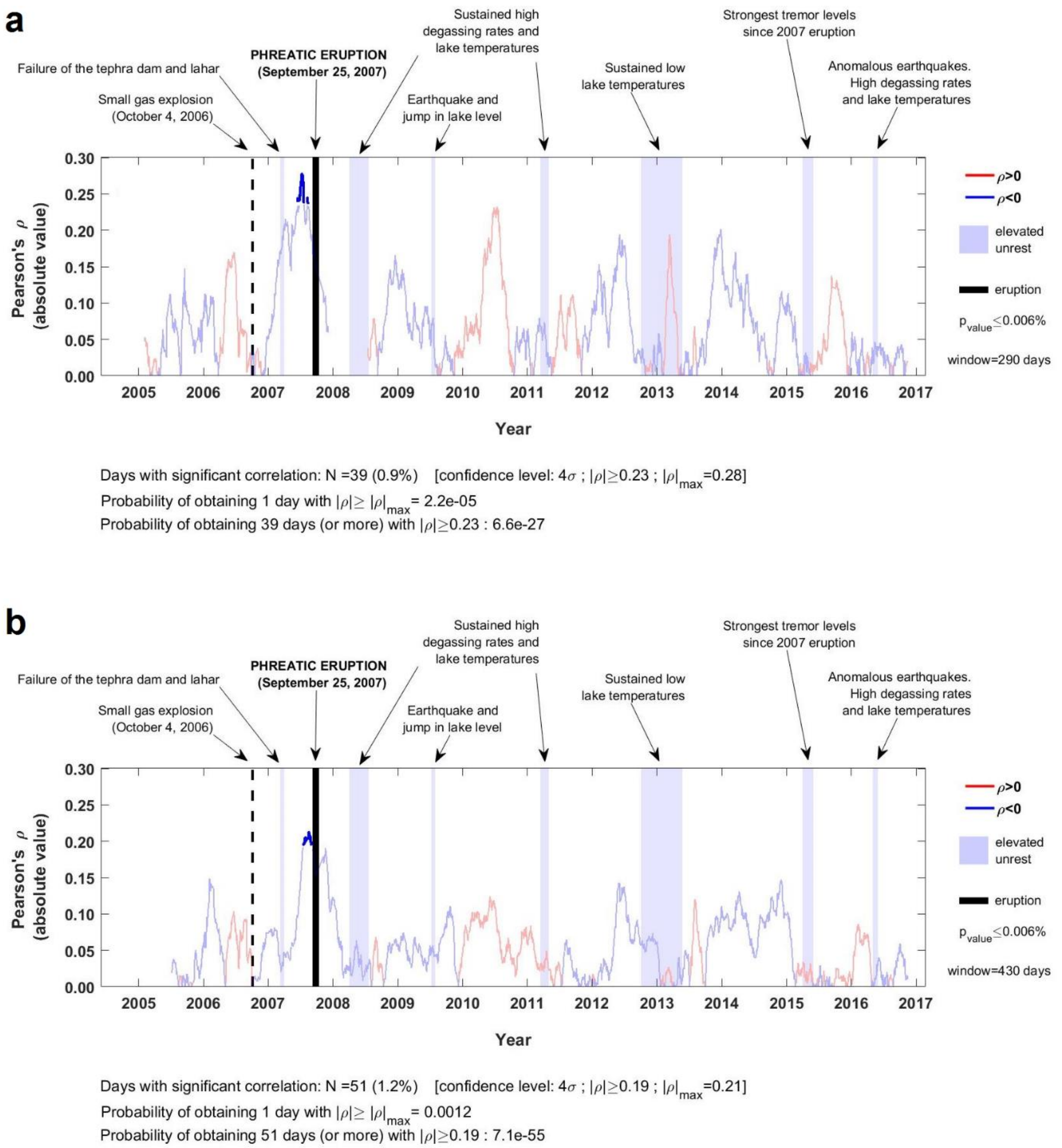


**Supplementary Figure S6. Luni-seismic correlation obtained for Ruapehu volcano with different backward windows. a,** Correlation with backward window size of 290 days. **b,** Correlation with backward window size of 430 days. Significant correlation emerges prior to the 2007 phreatic eruption for this range of windows. See caption of Figure 2 for further explanations of the panels.

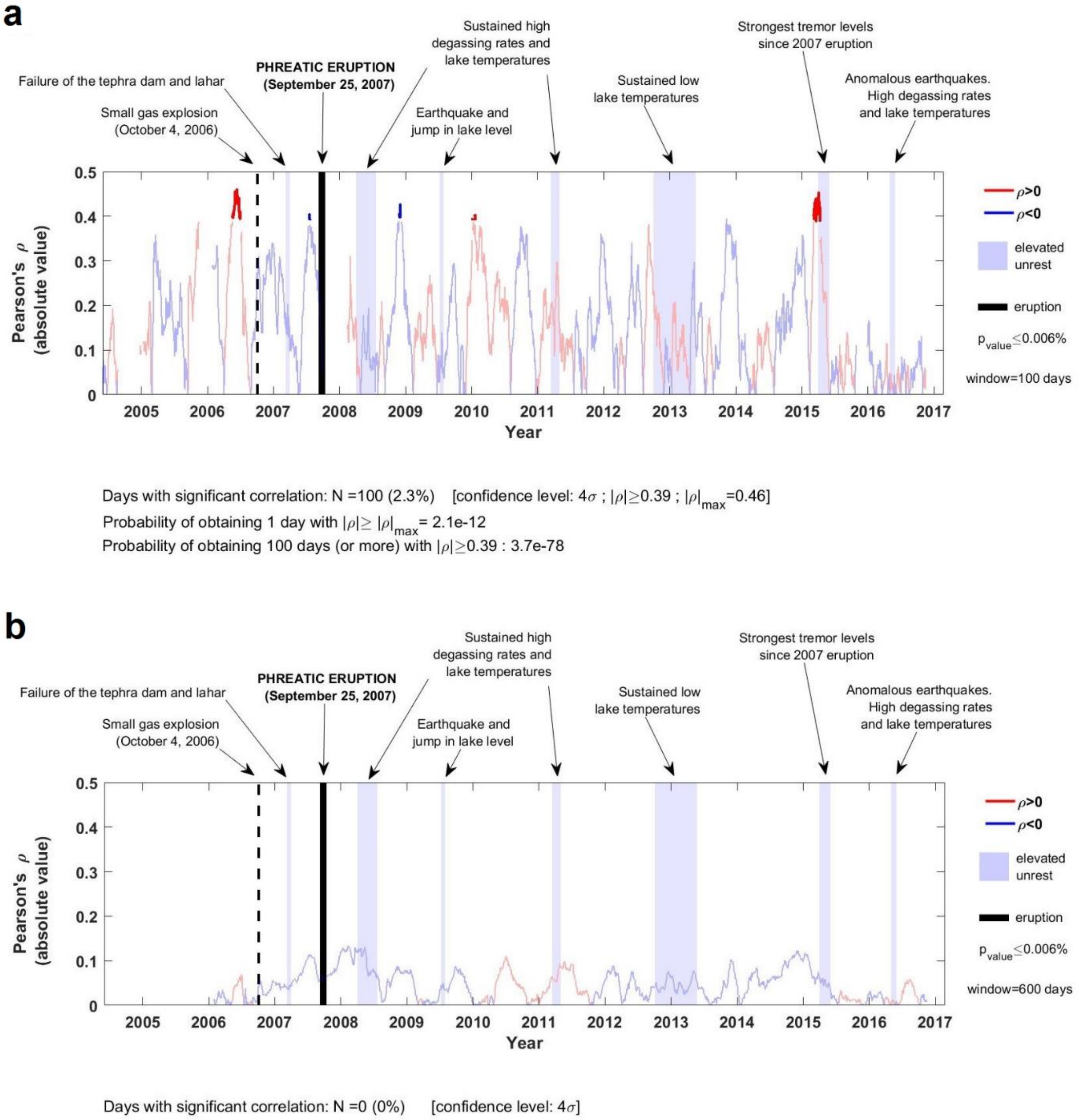


**Supplementary Figure S7. Luni-seismic correlation obtained for Ruapehu volcano with different backward windows. a,** Correlation with backward window size of 100 days. Short-term peaks of correlation emerge sporadically, probably due to the instability introduced by the small sample size and the noisy nature of the data[34]. **b,** Correlation with backward window size of 600 days. No sensitivity to lunar cycles appears in these timescales, probably because the mechanism behind the seismic response to fortnightly tides does not operate on so large timescales. See caption of Figure 2 for further explanations of the panels.

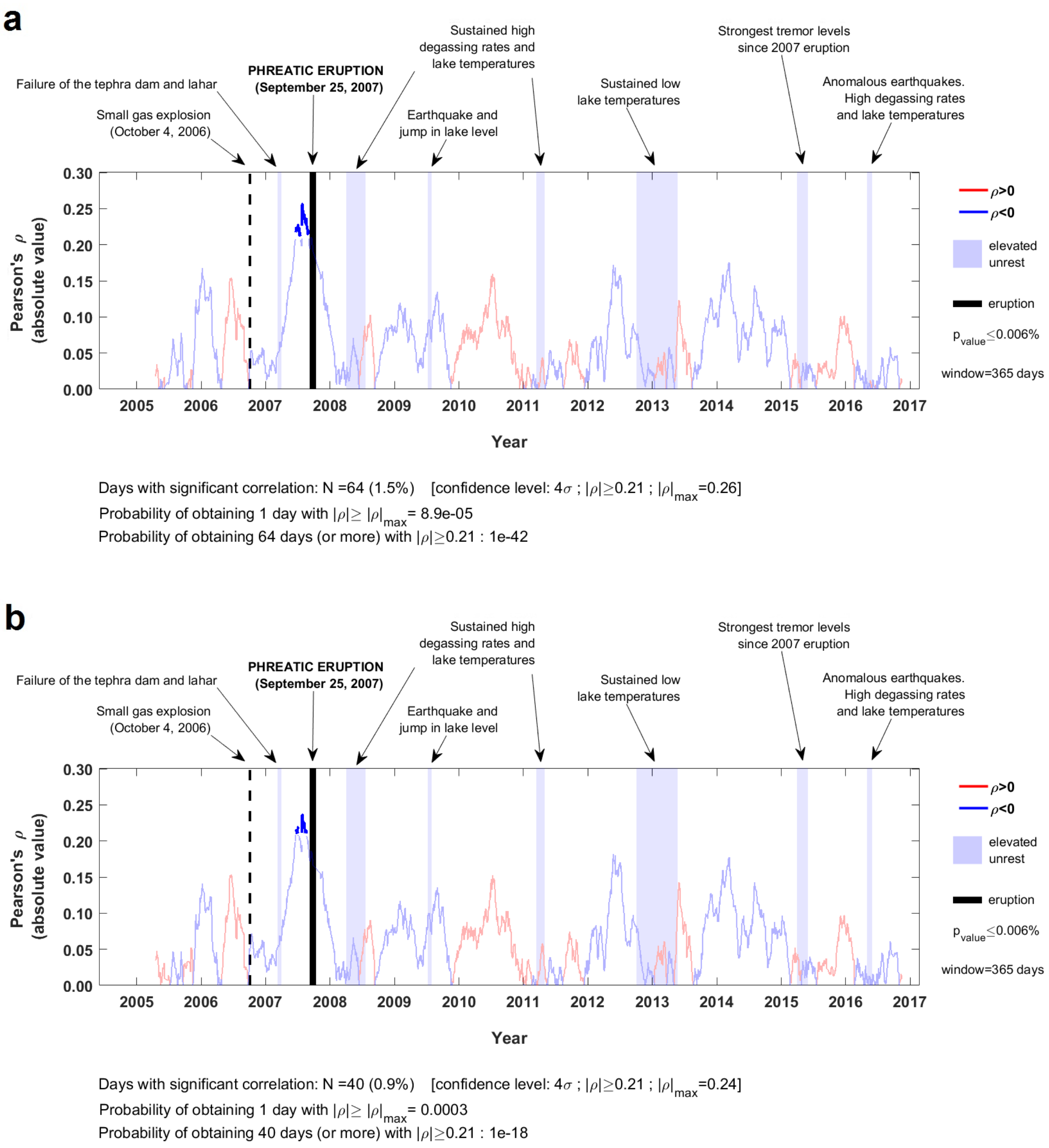


**Supplementary Figure S8. Influence of the selection method of the daily median seismic amplitude on the luni-seismic correlation** (see details in Methods, Section 1)**. a**, The daily median seismic amplitude is chosen using moving windows of 30 s. **b**, The daily median seismic amplitude is chosen using moving windows of 1800 s. Our results are essentially the same for a wide range of moving windows. See caption of Figure 2 for further explanations of the panels.

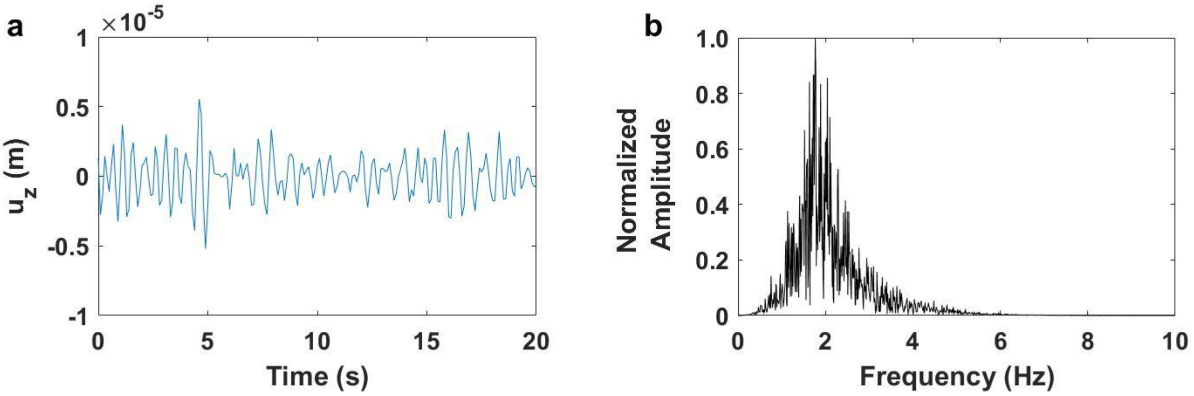


**Supplementary Figure S9. Example of synthetic volcanic tremor generated to explore the correlation between lunar cycles and seismic amplitude** (using the model proposed by Ref. 35)**. a,** Synthetic ground displacement ($u_z$) with time. **b,** Synthetic amplitude spectrum of the ground displacement. We use realistic values for the different parameters involved in the model (see details in the caption of Figure 4).

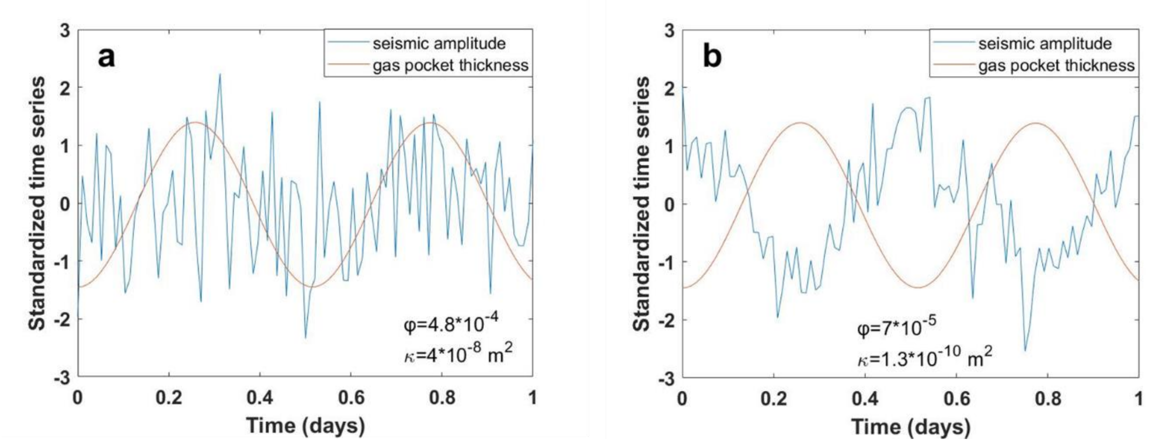


**Supplementary Figure S10. Daily variations of the gas pocket thickness and seismic amplitude for two different permeabilities.** **a,** Scenario with high porosity and permeability; seismic amplitude is slightly sensitive to the daily variations of the gas pocket thickness. **b,** Scenario with low porosity and permeability; seismic amplitude is highly sensitive to the daily variations of the gas pocket thickness. We use realistic values for the different parameters of the model (see details in Methods, Section 3). The values of porosity and permeability are realistic for permeable flow controlled by cracks and channels[35].

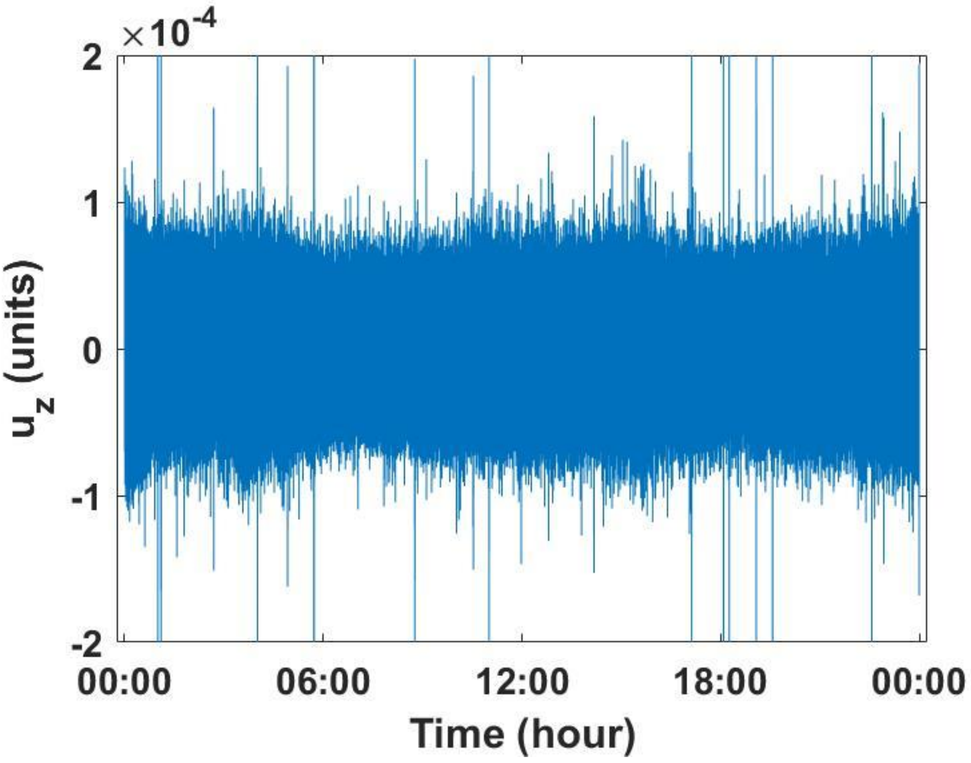


**Supplementary Figure S11. Example of semi-diurnal modulation of the seismic amplitude at Ruapehu.** This case can be detected by eye inspection, and corresponds to the seismic data recorded on March 19, 2007 (i.e., within the time period where Ruapehu was sensitive to lunar cycles).

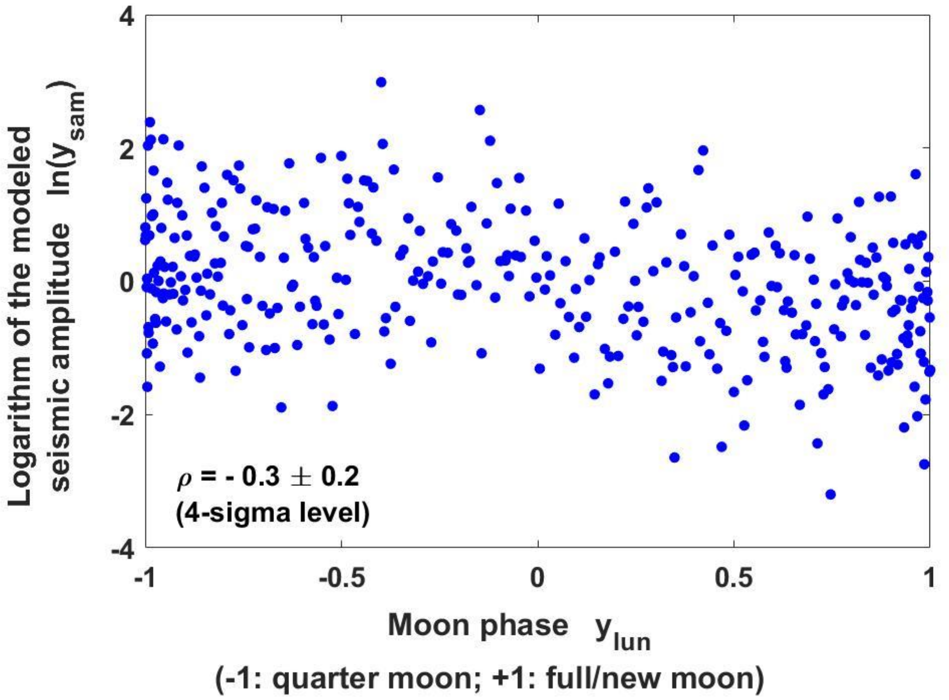


**Supplementary Figure S12. Example of modeled seismic amplitude vs Moon phase for a case of high correlation (in absolute value; $\rho = -0.38$).** This is obtained assuming cap porosity $\varphi = 1.2\%$. The values of the rest of parameters of the model are given in Methods, Section 3. Note that these graphs obtained with our model are comparable to those obtained with the natural data (Fig. 2b, 2c).

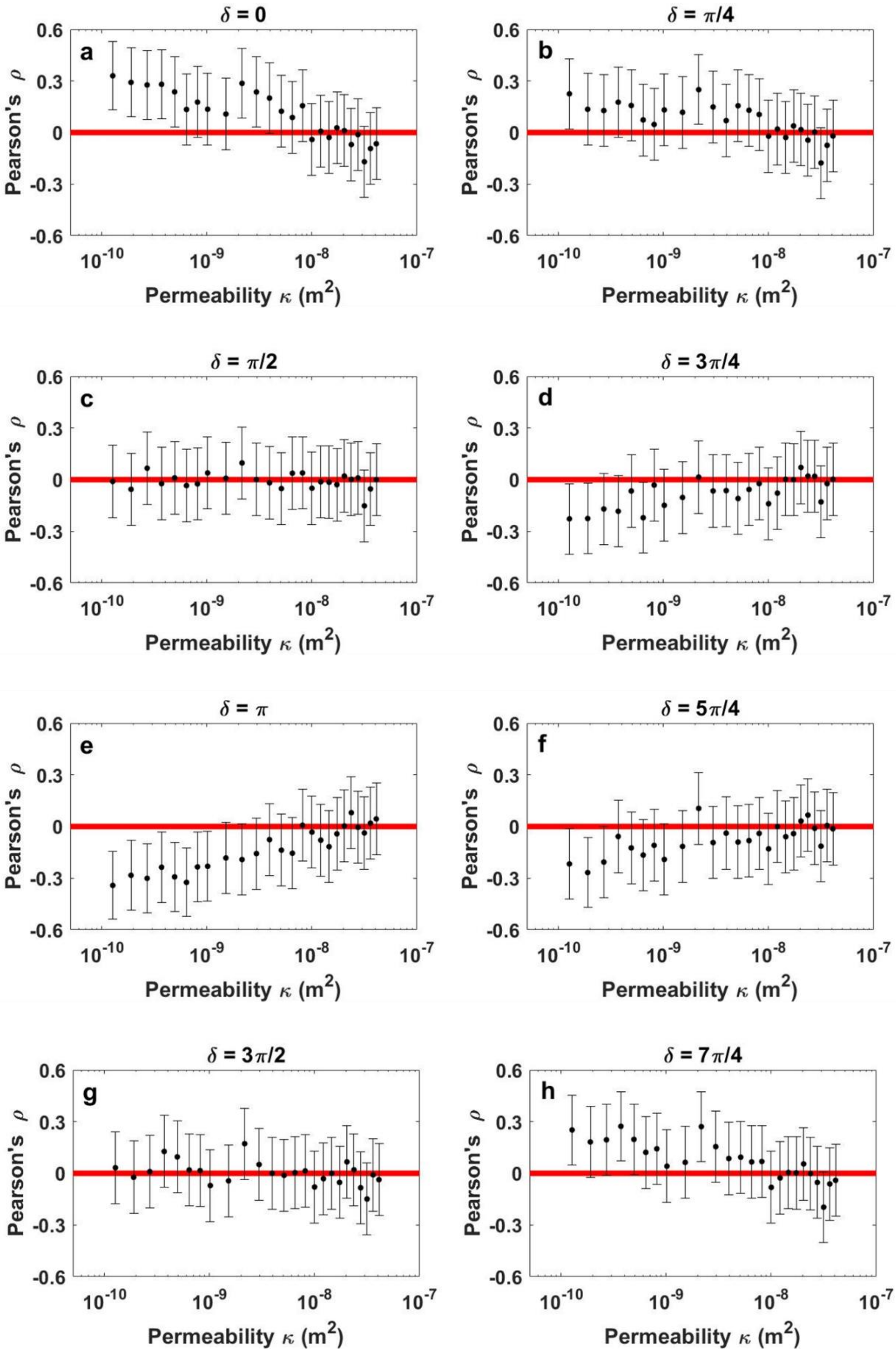


**Supplementary Figure S13. Pearson coefficient (*ρ*) between lunar cycles and the modelled seismic amplitude for different values of cap permeability (*κ*) and phase shift *δ*.** See caption of Figure 4 for further explanations of the panels.
